# The prognosis value of CONUT and SIS score for recurrent or metastatic esophageal squamous cell carcinoma patients treated with second-line immunotherapy

**DOI:** 10.3389/fonc.2023.1167625

**Published:** 2023-06-14

**Authors:** Xiao-Han Zhao, Wen-Bin Shen, Duo Wang, He-Song Wang, Chun-Yang Song, Wen-Zhao Deng

**Affiliations:** ^1^ Department of Radiation Oncology, the Fourth Hospital of Hebei Medical University, Shijiazhuang, China; ^2^ Hebei Key Laboratory of Animal Physiology, Biochemistry and Molecular Biology, College of Life Sciences, Hebei Normal University, Shijiazhuang, China

**Keywords:** esophageal squamous cell carcinoma, second-line treatment, immune checkpoint inhibitors, controlling nutritional status, systemic inflammation score, prognosis

## Abstract

**Objective:**

To investigate the predictive value of Controlling Nutritional Status (CONUT) score and systemic inflammation (SIS) score in the prognosis, short-term efficacy, and immune-related side effects of patient with recurrent or metastatic esophageal squamous cell carcinoma (R/M ESCC) receiving immunotherapy as second line therapy combined with or without radiotherapy.

**Methods:**

Forty-eight patients with R/M ESCC who received second-line therapy with Camrelizumab were retrospectively studied. They were divided into the high and low score groups according to the CONUT and SIS score. Univariate and multivariate analyses were used to analyze factors that might affect patient prognosis and the effects of different CONUT score and SIS on the short-term efficacy and immune-related toxic and side effects of patients.

**Results:**

The 1- and 2-year overall survival (OS) and progression-free survival (PFS) rates were 42.9% and 22.5%, and 29.0% and 5.8%, respectively. The CONUT score ranged from 0 to 6 (3.31 ± 1.43), whereas the SIS score ranged from 0 to 2 (1.19 ± 0.73). Multivariate analysis showed that treatment related toxicity, number of cycles of Camrelizumab used, short-term effect and SIS score were independent prognostic factors for OS (*P*=0.044, 0.021, 0.021, 0.030, respectively), whereas SIS and CONUT scores were independent prognostic factors for PFS (P=0.005, 0.047, respectively). Patients with low CONUT/SIS score had a low incidence rate of immune-related adverse reactions (X^2 = ^9.735, 5.693; *P*=0.002, 0.017) and better short-term efficacy (X^2 = ^4.427, 7.438; *P*=0.035, 0.006).

**Conclusion:**

R/M ESCC patients with low CONUT/SIS score have better prognosis, higher objective response rate, lower incidence of immune-related toxic and side effects after receiving immunotherapy as second-line therapy. CONUT scores and SIS scores may be reliable prognostic indicators for patient receiving immunotherapy as second-line therapy for R/M ESCC.

## Introduction

Esophageal cancer (EC) is one of the most prevalent and malignant tumors worldwide. Esophageal squamous cell carcinoma (ESCC) is the globally predominant pathological type of EC, which accounts for 90% of all ECs in China (1). Esophageal cancer is often diagnosed at an advanced stage, although the treatment modalities of ESCC have greatly improved, its 5-year survival rate is still low because of high local recurrence and distant metastasis (2).

The best treatment modality for patient with recurrent or metastatic ESCC after first-line radical treatment remains controversial. Recently, various targeted drugs and immune checkpoint inhibitors (ICIs) have achieved certain efficacy in the treatment of ESCC, with the latter greatly promoting the prognosis of ESCC patients (3–5). The ICIs always applied combined with chemoradiotherapy, which sometimes lead to serious side effects including pneumonia or cardiovascular injury. Thus, developing ICIs that can target ESCC and exploring reliable indicators to predict the treatment outcomes of ICIs are important. Previous studies have shown that systemic inflammation and malnutrition are significant factors influencing prognosis in patients with malignant tumors (6, 7). The Controlling Nutritional Status (CONUT) score consists of serum albumin (ALB), peripheral lymphocyte (PLM) count, and total cholesterol (TC). Meanwhile, the systemic inflammation (SIS) score comprises ALB levels and lymphocyte-to-monocyte ratio (LMR). The validity of these tools in predicting prognosis and treatment-related complications has been reported in various cancers including ESCC (8–12). The components of COUNT and SIS reflect the inflammatory responses and nutritional status of human body, which might correlate with the treatment efficacy and prognosis for ESCC receiving immunotherapy. There were already studies showing the prognostic value of CONUT for ESCC patients who were treated with neoadjuvant immunotherapy (13). However, the prognostic value of the CONUT and SIS score in patients with recurrent and/or metastatic ESCC (R/M ESCC) after first-line treatment failure treated with immunotherapy has not been investigated. The main objective of this study was to investigate the clinical significance of the CONUT and SIS score in patient with R/M ESCC treated with immunotherapy as second-line treatment.

## Materials and methods

### Patient selection

A cohort of patients treated at the Radiotherapy Department of the Fourth Hospital of Hebei Medical University from January 2018 to March 2021 were retrospectively collected. The inclusion criteria were as follows: (1) patients aged 18–80 years old, (2) histologically or cytologically diagnosed with ESCC, (3) experienced recurrence or metastasis after first-line treatment, (4) receiving Camrelizumab alone or in combination with chemo-/radiotherapy for more than two cycles as second-line treatment, (5) having Eastern Cooperative Oncology Group (ECOG) Performance Status score between 0 and 2, and (6) having accessible peripheral hematologic parameters and complete imaging data with at least one measurable lesion. The exclusion criteria were as follows: (1) ICIs were used during first-line treatment; (2) combination of malignancies other than ESCC; (3) patients with acute inflammatory, hematologic, or autoimmune diseases; and (4) incomplete clinical or pathological data. Finally, 48 patients were included in this study. Because of the retrospective nature of this study, informed consent from patients was waived. Ethical approval was obtained from the Ethics Committee of the Fourth Hospital of Hebei Medical University before the start of the study.

### Data collection

General clinical information, including age, gender, ECOG score, pathological type, first-line treatment modality, interval between recurrence/metastasis and first-line treatment, second-line treatment regimen, number of Camrelizumab cycles, efficacy of Camrelizumab after 3 months of treatment, incidence of side effects associated with ICIs, and peripheral blood information (including ALB, PLM, TC, and monocyte [MC] values within 1 week prior to ICI treatment) was collected.

### Outcome

Assessment was based on immune-related Response Evaluation Criteria In Solid Tumor (irRECIST) and confirmed by two independent radiologists through computed tomography (CT) or magnetic resonance imaging (MRI). To avoid the effect of pseudo-progression of immunotherapy, the efficacy of ICIs at 3 months after treatment was evaluated. The short term effect was classified as complete response (CR), partial response (PR), stable disease (SD), and progressive disease (PD). The overall response rate (ORR) was calculated based on the percentage of patients who achieved CR and PR. The disease control rate (DCR) was calculated based on the percentage of patients who achieved CR, PR, and SD. Treatment-related toxic and side effects were evaluated using the Common Terminology Criteria for Adverse Events version 5.0. Immune-related adverse events (irAEs) were used to record toxic and side effects associated with immunotherapy.

### Follow-up

Follow-up was performed for patients from the beginning of treatment until the date of death from all causes or until the last approachable follow-up. It was conducted every two to three cycles during treatment and every 1–3 months thereafter, mainly through visit, telephone interview, or Internet. Immune-related hematological tests; imaging and pathological examinations such as ultrasound, CT, or MRI; and cytological examination were mainly performed. The final follow-up date was June 30, 2022.

### Statistical analysis

SPSS 26.0 and GraphPad Prism 9.3.1 software were applied for statistical analysis. The enumeration data were expressed as a rate or constituent ratio and statistically analyzed using X^2^ test or Fisher test; The Kaplan–Meier survival curve was used for the comparison of overall survival (OS) and progression-free survival (PFS). Multifactorial analysis was performed using the Cox regression model for factors with *P*<0.1 in univariate analysis, LR backward method was utilized. The corresponding 95% confidence intervals (CIs) were calculated. Receiver operating characteristics (ROC) curve analyses were utilized to calculate area under the curve, statistical significance was considered at *P*<0.05. PFS was defined as the time from the beginning of treatment to imaging-documented disease progression, death, or last follow-up. OS was defined as the time from the first treatment with ICIs to death or last patient contact.

## Results

### Clinical characteristics

Forty-eight patients with R/M ESCC after first-line treatment failure (including 32 males and 16 females) met the inclusion criteria. The median age was 65 years old. Twenty-two patients were receiving radical surgical resection as the first-line treatment modality. Meanwhile, 18 and 8 patients were receiving radical chemoradiotherapy and radical radiotherapy as first-line treatment, respectively. Among the patients, 31 cases experienced locoregional recurrence, 3 cases experienced distant metastasis, and 14 cases experienced both after first-line treatment failure. The ECOG scores were 0, 1, and 2 for 5, 18, and 25 cases, respectively. The PFS for first-line treatment was 3–114 months, of which 15 and 33 cases had a PFS of ≤12 and >12 months, respectively. Camrelizumab was used in combination with chemoradiotherapy for 26 patients, chemotherapy for 14 patients, radiotherapy for 4 patients and as monotherapy for 4 patients. Intensity-modulated radiation therapy was applied to irradiate the involved field of malignancy, with an equivalent biological dose of 45–66 Gy, 1.8–5.0 Gy per fracture, and 2.0 Gy per fracture as the median.

### Results of CONUT score and SIS analysis

In the whole group, the ALB value ranged from 3.14 to 4.74 g/dl (4.10 ± 0.35), the lymphocyte count was 230–2060/mm^3^ (971.3 ± 460.16), the TC was 129.7–438.0 mg/dl (170.13 ± 44.36), and the LMR ranged from 0.15 to 6.94 (3.09 ± 1.72). The CONUT score ranged from 0 to 6 (3.31 ± 1.43), with 1, 4, 10, 15, 12, 1, and 5 patients having a score of 0, 1, 2, 3, 4, 5, and 6, respectively. The SIS ranged from 0 to 2 (1.19 ± 0.73), with 9, 21, and 18 patients having a score of 0, 1, and 2, respectively (see **Table 1** for details).

**Table 1:** The CONUT and SIS scoring criteria.

CONUT scoring criteria			SIS scoring criteria	
Variables	Range	Score	Variables	Score
Albumin(g/dL)	≥3.50 3.00-3.49 2.50-2.99 <2.50	0 2 4 6	Albumin(g/dL)≥4.0 and LMR≥4.44	0
Total cholesterol(mg/dL)	≥180 140-180 100-139 <100	0 1 2 3	Albumin(g/dL)<4.0 or LMR<4.44	1
Absolute lymphocyte count(*/mm3*)	≥1600 1200-1599 800-1199 <800	0 1 2 3	Albumin(g/dL)<4.0 and LMR<4.44	2

The patients were divided into two groups according to the CONUT and SIS score to explore their relationship with general clinical characteristics and treatment outcomes. The results showed a significant difference between the use of chemoradiotherapy as combination therapy and patients with CONUT score ≤3 and >3 (see **Table 2** for details).

**Table 2:** The constitution of clinicopathologic features in different CONUT and SIS groups.

Clinicopathologic features	N	CONUT		X^2^	*P*	SIS		X^2^	*P*
		≤3	>3			≤1	>1		
Gender									
Male	32	18	14	1.600	0.206	22	10	1.600	0.206
Female	16	12	4			8	8		
Age (year)									
≤65	24	16	8	0.356	0.551	14	10	0.356	0.551
>65	24	14	10			16	8		
ECOG									
2	23	16	7	0.941	0.332	15	8	0.139	0.709
0-1	25	14	11			15	10		
First line treatment modality									
Surgery	22	15	7	0.559	0.454	17	5	3.782	0.052
None-surgery	26	15	11			13	13		
Recurrence type									
Local regional	35	21	14	0.345	0.557	20	15	1.582	0.208
others	13	9	4			10	3		
Interval after treatment failure									
≤12 month	15	9	6	0.058	0.809	8	7	0.567	0.451
>12month	33	21	12			22	11		
**Camrelizumab combined modality**								2.009	0.156
Chemoradiotherapy	25	21	4	10.290	0.001*	18	7		
Others	23	9	14			12	11		
**Number of cycles** **of immune-therapy used**				2.009	0.156			3.527	0.060*
<6 cycles	21	18	7			10	11		
≥6 cycles	27	12	11			20	7		

### Short-term efficacy and prognosis

**Figure 1:**
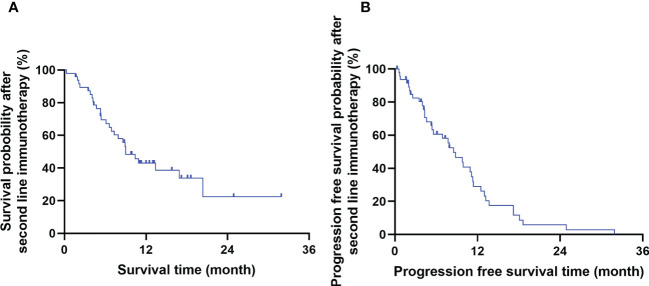
The overall survival time **(A)** and progression free survival time **(B)** of the whole cohort.

The short-term efficacy of Camrelizumab was evaluated 3 months after treatment completion. The ORR and DCR were 35.4% (17/48) and 91.7% (44/48), respectively. 0, 17, 27, and 4 cases achieved CR, PR, SD, and PD, respectively. The 1- and 2-year OS and PFS rates after immunotherapy were 42.9% and 22.5%, and 29.0% and 5.8%, respectively. The median OS and PFS were 9.0 months (95% CI: 6.4–11.7) and 8.5 months (95% CI: 1.5–5.6), respectively. The survival curve is shown in **Figure 1**.

Univariate analysis showed that Camrelizumab combined with chemoradiotherapy, toxic and side effects, Number of cycles of Camrelizumab used, short-term efficacy, SIS, and CONUT scores were significant factors influencing OS (X^2 = ^3.821, 7.529, 13.144, 8.112, 14.354, 11.623; *P*=0.047, 0.006, 0.000, 0.004, 0.000, 0.001, respectively). Meanwhile, Camrelizumab combined with chemoradiotherapy, number of cycles of Camrelizumab used, short-term efficacy, SIS and CONUT scores were significant factors influencing PFS (X^2 = ^4.426, 6.100, 3.803, 14.812, 10.018; *P*=0.035, 0.014, 0.048, 0.000, 0.002, respectively).

**Figure 2:**
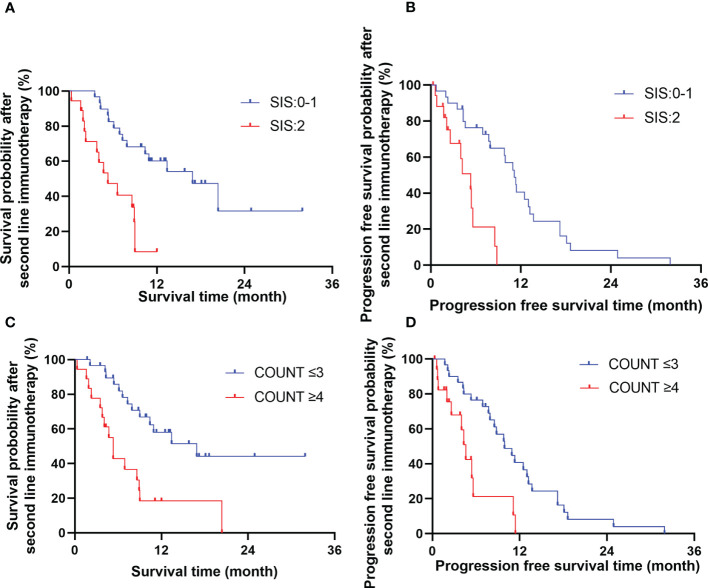
The effects of SIS **(A, B)** and COUNT **(C, D)** score on overall survival time and progression free survival time.

Multivariate analysis showed that treatment related toxicity, number of cycles of Camrelizumab used, short-term effect and SIS score were independent prognostic factors for OS (*P*=0.044, 0.021, 0.021, 0.030, respectively), whereas SIS and CONUT scores were independent prognostic factors for PFS (*P*=0.005, 0.047, respectively). See **Table 3** for details. The effects of CONUT and SIS score on OS and PFS were further analyzed using the survival curve, as shown in **Table 4** and **Figures 2A–D**.

**Table 3:** Univariate and multivariate analysis of the whole cohort.

Clinicopathologic features	Univariate analysis		multivariate analysis	
	X^2^	*P*	HR(95%CI)	*P*
OS				
**Genger** (male/female)	0.096	0.757		
**Age** (≤65years old/65years old)	0.039	0.843		
**ECOG** (2/0-1)	0.007	0.935		
**First line treatment modality** (surgery/non-surgery)	2.309	0.203		
**Recurrence type** (Local regional/others)	0.244	0.622		
**Interval after treatment failure** (≤12 month/>12month)	2.614	0.106		
**Camrelizumab combined modality** (Chemoradiotherapy/Others)	3.821	0.047		
**Treatment related toxicity** (yes/no)	7.529	0.006	0.440(0.198-.0980)	0.044
**Number of cycles of Camrelizumab used** (<6 cycles/≥6 cybles)	13.144	0.000	0.341(0.137-0.847)	0.021
Short-term effects (PR/SD/PD)	8.112	0.004	3.174(1.186-8.497)	0.021
**SIS score** (≤1/>1)	14.354	0.000	2.749(1.091-6.930)	0.030
**CONUT score** (≤3/>3)	11.633	0.001		
PFS				
**Genger** (male/female)	0.095	0.757		
**Age** (≤65years old/65years old)	0.001	0.973		
**ECOG** (2/0-1)	0.474	0.492		
**First line treatment modality** (surgery/non-surgery)	2.104	0.716		
**Recurrence type** (Local regional/others)	0.068	0.565		
**Interval after treatment failure** (≤12 month/>12month)	0.001	0.980		
**Camrelizumab combined modality** (Chemoradiotherapy/Others)	4.426	0.035		
**Treatment related toxicity** (yes/no)	0.000	0.995		
**Number of cycles of Camrelizumab used** (<6cycles/≥6cybles)	6.100	0.014		
**Short-term effects** (PR/SD/PD)	3.703	0.054		
**SIS score** (≤1/>1)	14.812	0.000	3.784(1.479-9.677)	0.005
**CONUT score** (≤3/>3)	10.018	0.002	2.355(1.012-5.481)	0.047

**Table 4:** Survival analyses according to CONUT score and SIS score.

Index	OS(%)		Median(month)	95%CI	PFS(%)		Median(month)	95%CI
	1-year	2-year			1-year	2-year		
CONUT score								
≤3	44.1	–	16.9	7.17~26.56	40.5	8.1	9.9	6.59~13.21
>3	18.3	0	5.3	4.09~6.52	0	0	4.6	3.64~5.56
SIS score								
≤1	60.0	31.5	17.2	9.72~24.01	41.3	8.4	11.1	8.98~13.29
>1	8.5	–	5.5	2.07~8.53	0	0	5.0	3.33~7.34

### Toxic and side effects

There were 26 patients with possible treatment-related toxic and side effects. Among them, there were 6 cases of myelosuppression (1 case of grade II, 4 cases of grade III, and 1 case of grade IV), 6 cases of pneumonia (2 cases of grade II and 4 cases of grade III), 4 cases of hematemesis or hemoptysis, 4 cases of reactive cutaneous capillary endothelial proliferation (1 case of grade I, 3 cases of grade II), 1 case of grade II gastrointestinal reaction combined with grade III myelosuppression, 2 cases of thyroid dysfunction (all performed as hypothyroidism), 1 case of pneumonia with esophageal fistula (grade II), 1 case of hemolytic anemia, and 1 case of liver dysfunction (grade III). A total of 16 patients discontinued immunotherapy for various reasons, including 6 cases of pneumonia, 4 cases of hematemesis or hemorrhage, 2 cases of refusal, 1 case of thyroid dysfunction, 1 case of pneumonia with esophageal fistula, 1 case of hemolytic anemia, and 1 case of liver dysfunction. A total of 10 cases discontinued treatment due to toxicity of immunotherapy, see **Table 5** for details.

**Table 5:** Toxic and side effects of the whole cohort.

Toxic and side effects	grade I	grade II	grade III	grade IV
Myelosuppression	–	1	4	1
Pneumonia	–	2	4	–
Reactive cutaneous capillary endothelial proliferation	1	3	–	–
Liver dysfunction	–	–	1	–
Others	Hematemesis/Hemoptysis for 4, Thyroid dysfunction for 2, Hemolytic anemia for 1, grade II gastrointestinal reaction combined with grade III myelosuppression for 1, pneumonia with esophageal fistula for 1, hemolytic anemia for 1,			

### Prognostic value of CONUT and SIS

The patients were divided into two groups according to the CONUT and SIS score. Patients with CONUT score ≤3 were included in the CONUT low group, and those with CONUT score >3 were included in the CONUT high group. Patients with SIS ≤1 were included in the SIS low group, and those with SIS >1 were included in the SIS high group. The results showed that patients with low CONUT score had lower incidence of immune-related adverse reactions (X^2 = ^9.735, *P*=0.002) and better short-term effect (X^2 = ^4.427, *P*=7.438) than those with high CONUT score. While in the SIS group, a lower SIS score related to a low incidence of immune-related adverse reactions (X^2 = ^5.693, *P*=0.002) and improved short term effect (X^2 = ^7.438, *P*=0.006). See **Table 6** for details.

**Table 6:** Chi-square test of CONUT scores and SIS scores and treatment-related factors.

Index	N	CONUT score		X^2^	*P*	SIS score		X^2^	*P*
		≤3	>3			≤1	>1		
**Treatment related toxicity**				0.201	0.654			0.970	0.295
yes	26	17	9			18	8		
no	22	13	9			12	10		
**Immune-related adverse reactions**				9.735	0.002*			5.693	0.017*
yes	10	2	8			3	7		
no	38	28	10			27	11		
**Short-term effects**				4.427	0.035*			7.438	0.006*
PR	17	14	3			15	2		
SD/PD	31	16	15			15	16		

* means the P values were less than 0.05 and showed significant difference.

### Comparison of COUNT and SIS score with other hematology indexes

We collected Peripheral blood and performed Receiver operating characteristics (ROC) curve analyses of 1-year survival rate for associated hematology indexes that had been reported to been related to prognosis, including neutrophil, monocyte, lymphocyte, platelet, albumin (ALB), total cholesterol (TC), neutrophil to lymphocyte ratio (NLR), neutrophil to monocyte ratio (NMR), platelet to lymphocyte ratio (PLR), and prognostic nutritional index (PNI), the results were shown in **Table 7**. It was shown that COUNT score and SIS scores had significant power for predicting prognosis, besides, the area under the curve for COUNT score and SIS score were higher than other hematology indexes.

**Table 7:** Comparison of COUNT and SIS score with other haematology indexes.

Haematology indexes	Area under the curve	*P*	95% CI
COUNT score	0.682	0.045*	0.519~0.845
SIS score	0.708	0.021*	0.550~0.867
Neutrophil	0.589	0.323	0.412~0.766
Monocyte	0.634	0.125	0.463~0.805
Lymphocyte	0.549	0.087	0.377~0.720
Platelet	0.632	0.146	0.462~0.801
Albumin (ALB)	0.587	0.337	0.415~0.759
Total cholesterol (TC)	0.642	0.088	0.479~0.805
Neutrophil to lymphocyte ratio (NLR)	0.606	0.089	0.432~0.781
Neutrophil to monocyte ratio (NMR)	0.608	0.089	0433~0.783
Platelet to lymphocyte ratio (PLR)	0.593	0.090	0.417~0.769
Prognostic nutritional index (PNI)	0.400	0.091	0.222~0.579

## Discussion

To the best of our knowledge, this is the first study to examine the association of nutritional immune-inflammatory indexes with the prognosis, efficacy, and toxicity of ICIs as second-line therapy in patient with R/M ESCC.

In this study, for R/M ESCC patient, the OS and PFS were significantly better in low CONUT score (≤3) group compared with those in high CONUT score (>3) group, Similarly, SIS score(≤ 1) group had significantly prolonged OS and PFS compared with SIS score (>1) group. Multivariate analysis showed that SIS was an independent predictors of OS and PFS, besides, COUNT was an independent predictors for PFS. Therefore, CONUT and SIS can be used as biomarkers to predict the prognosis of patient with R/M ESCC receiving ICIs as second-line therapy.

CONUT and SIS mainly comprise peripheral blood indexes including serum ALB, PLM, TC, and MC. Serum ALB mainly reflects the ability of the body to synthesize protein, serum TC reflects the ability to metabolize lipids, and PLM and MC reflect human immune function (14, 15). A subjective assessment of patients’ nutritional immune-inflammatory status is inaccurate and complex. Thus, CONUT/SIS can provide an easier and more objective method to comprehensively evaluate the nutritional immune-inflammatory status of patients. High CONUT/SIS can not only reflect malnutrition, but also reflect systemic inflammation and impaired immune response. In this study, we retrospectively analyzed the prognostic value of CONUT and SIS and found that patients with low CONUT/SIS score had significantly lower incidence of ICI-related toxic and side effects than those with high CONUT/SIS. Moreover, the low CONUT/SIS group had significantly better short-term efficacy compared with the high CONUT/SIS group. To the best of our knowledge, no studies have used CONUT and SIS in predicting ICI toxicity and short-term efficacy for patients with R/M ESCC receiving ICIs as second-line therapy. This study found that CONUT and SIS score had a clinical value in predicting the effect and possible toxic and side effects of patient with R/M ESCC receiving second-line immunotherapy, which had better power for predicting prognosis than other hematology indexes like albumin, total cholesterol and prognostic nutritional index (PNI). Thus, the utility of CONUT and SIS before second-line immunotherapy should be further investigated.

Previous studies mainly focused on the prognostic value of CONUT score for patient with ESCC receiving esophagectomy. Takagi et al. (16) conducted a meta-analysis on the effect of preoperative CONUT score on postoperative prognosis in patients with EC receiving esophagectomy. A total of 952 patients were included. The results showed that the CONUT score was significantly correlated with OS, cancer-specific survival, and relapse-free survival (hazard ratio [HR]=2.51, 2.60, 2.08, *P*<0.001, respectively). Thus, the CONUT score is an independent predictor of prognosis in patients undergoing esophagectomy.

The relationship between CONUT score and patients with EC receiving ICIs has only been reported recently. For example, Chang et al. (17) retrospectively analyzed the clinical data of 69 patients with advanced EC treated with ICI and evaluated the relationship between nutritional immune-inflammatory indexes including CONUT score and prognosis. The results showed that high CONUT score was significantly correlated with poorer PFS and OS compared with low CONUT score. The authors believe that the CONUT score can be used as an effective index to predict the prognosis of patients with EC treated with ICIs. In addition, Feng et al. (18) confirmed the predictive value of the CONUT score in predicting recurrence in patients with EC who received neoadjuvant immunochemotherapy (nICT). They retrospectively analyzed 216 patients with ESCC who received nICT and found that patients with high CONUT score (≥3) had a significantly higher recurrence rate (24.2% versus 9.3%, *P*=0.004) and lower 1-year DFS (75.8% versus 90.7%) compared to those with low CONUT score (≤2) (*P*=0.004). Multivariate analysis showed that CONUT score was an independent predictor of DFS (HR=2.221, *P*=0.033). Although these studies did not focus on R/M ESCC, they proved the value of the CONUT score in predicting the prognosis of patients with EC receiving immunotherapy to some extent.

SIS is a new grading system based on serum ALB and LMR. According to literature, no studies used SIS to evaluate the efficacy of immunotherapy for EC. Chang (19) first used SIS in predicting the prognosis of patients with renal clear cell carcinoma, which was based on preoperative serum ALB and median LMR. SIS also showed prognostic value in patients with lung cancer treated with ICIs (20).

Studies of SIS in ESCC mainly focus on patients receiving esophagectomy. For example, Aoyama (21) reported that the 3- and 5-year OS rates of patients with low SIS (≤1) and high SIS (2) were 61.9% and 33.3%, and 52.4% and 26.6%, respectively (P<0.001). The incidence of postoperative grade 2 anastomotic leakage in the high SIS group was 61.5%, whereas that in the low SIS group was only 30.3%. The authors believed that SIS may be an important prognostic factor for patients with ESCC after esophagectomy. Nomoto (10) retrospectively analyzed 509 patients after ESCC. The results showed that the 3-year OS rates of patients with an SIS of 0, 1, and 2 were 84.1%, 74.6%, and 57.3%, respectively (*P*<0.001). Thus, SIS could be an independent risk factor for predicting OS (HR=1.76, *P*=0.013). Meanwhile, SIS was found to be more sensitive than other nutritional immunological prognostic factors (e.g., CONUT, systemic immune-inflammation index, and neutrophil–lymphocyte ratio), which could be used as a promising prognostic factor for patients with ESCC after surgery.

Malnutrition and nutritional deficiency are common problems for patients with malignant tumors, and most patients gradually progress to cachexia, which is significantly associated with treatment-related toxic effects and influences tolerance and efficacy of therapy, thus lowering patients’ quality of life and survival time (22). At present, there are many tools for screening patients with high risk of malnutrition. However, a precise assessment of the nutritional status of patients with malignant tumors is still needed (23, 24). The CONUT score and SIS are related to the immunonutrition and inflammatory state of patients. Previous studies indicated that the CONUT score was not only related to the incidence of postoperative complications in patients with gastrointestinal cancer (16), but also significantly related to the recurrence rate of patients with EC after surgery (18). While SIS was significantly related to the occurrence of pneumonia and anastomotic leakage in patients after esophagectomy (21), the CONUT and SIS score before second-line immunotherapy were significantly correlated with toxicity and short-term efficacy, indicating that these two indexes can predict the tolerance and response of patients receiving ICIs, so as to guide clinicians to develop treatment programs according to the individual situation of patients.

The limitations of this study are as follows: first, this study is limited by its retrospective nature and small sample size, there are some confounding factors like different immunotherapy combined modalities, which may affect the robustness of the results. Larger prospective randomized clinical trials are needed to draw the final conclusion. Second, our study was limited to patient with ESCC and may not be representative for patient with esophageal adenocarcinoma. In addition, the benefits and toxicities of ICI treatment for R/M ESCC are affected by several factors, and there may be some unknown and underlying factors that were not included in our analysis.

In conclusion, this study indicated that patient with R/M ESCC who have low CONUT/SIS score before second-line treatment with ICIs have better short-term efficacy and prognosis compared to those with high CONUT/SIS score. CONUT and SIS can be used as biomarkers to predict the prognosis of patient with R/M ESCC receiving second-line immunotherapy, thereby helping to guide clinicians to make better treatment decisions.

## Data availability statement

The raw data supporting the conclusions of this article will be made available by the authors, without undue reservation.

## Ethics statement

The studies involving human participants were reviewed and approved by the Ethics Committee of the Fourth Hospital of Hebei Medical University. The study was conducted in accordance with the Declaration of Helsinki (as revised in 2013). Individual consent for this retrospective analysis was waived.

## Author contributions

(I) Conception and design: X-HZ. (II) Administrative support: W-BS. (III) Provision of study materials or patients: H-SW, W-ZD. (IV) Collection and assembly of data: X-HZ, C-YS. (V) Data analysis and interpretation: W-BS, X-HZ, DW. (VI) Manuscript writing: All authors, (VII) Final approval of manuscript: All authors. All authors contributed to the article and approved the submitted version.
