# Corrigendum: The prognosis value of CONUT and SIS score for recurrent or metastatic esophageal squamous cell carcinoma patients treated with second-line immunotherapy

**DOI:** 10.3389/fonc.2023.1347301

**Published:** 2023-12-12

**Authors:** Xiao-Han Zhao, Wen-Bin Shen, Duo Wang, He-Song Wang, Chun-Yang Song, Wen-Zhao Deng

**Affiliations:** ^1^ Department of Radiation Oncology, the Fourth Hospital of Hebei Medical University, Shijiazhuang, China; ^2^ Hebei Key Laboratory of Animal Physiology, Biochemistry and Molecular Biology, College of Life Sciences, Hebei Normal University, Shijiazhuang, China

**Keywords:** esophageal squamous cell carcinoma, second-line treatment, immune checkpoint inhibitors, controlling nutritional status, systemic inflammation score, prognosis

**Incorrect Affiliation**

In the published article, there was an error in affiliation 1. The previous affiliation stated “Department of Radiation Oncology, the Forth Hospital of Hebei Medical University, Shijiazhuang, China,”. The corrected affiliation is: “Department of Radiation Oncology, the Fourth Hospital of Hebei Medical University, Shijiazhuang, China,”.

The authors apologize for this error and state that this does not change the scientific conclusions of the article in any way. The original article has been updated.

